# Effects of low-dose pirfenidone on survival and lung function decline in patients with idiopathic pulmonary fibrosis (IPF): Results from a real-world study

**DOI:** 10.1371/journal.pone.0261684

**Published:** 2021-12-23

**Authors:** Eung Gu Lee, Tae-Hee Lee, Yujin Hong, Jiwon Ryoo, Jung Won Heo, Bo Mi Gil, Hye Seon Kang, Soon Seog Kwon, Yong Hyun Kim

**Affiliations:** 1 Division of Pulmonary, Allergy and Critical Care Medicine, Department of Internal Medicine, Bucheon St. Mary’s Hospital, College of Medicine, The Catholic University of Korea, Seoul, Republic of Korea; 2 Department of Statistics and Data Science, Yonsei University, Seoul, South Korea; 3 Division of Pulmonary, Critical Care and Sleep Medicine, Department of Internal Medicine, Eunpyeong St. Mary’s Hospital, College of Medicine, The Catholic University of Korea, Seoul, Republic of Korea; 4 Department of Radiology, Bucheon St. Mary’s Hospital, College of Medicine, The Catholic university of Korea, Seoul, Republic of Korea; Vanderbilt University Medical Center, UNITED STATES

## Abstract

**Background:**

Idiopathic pulmonary fibrosis (IPF) is a chronic, progressive fibrosing interstitial pneumonia of unknown etiology. In several randomized clinical trials, and in the clinical practice, pirfenidone is used to effectively and safely treat IPF. However, sometimes it is difficult to use the dose of pirfenidone used in clinical trials. This study evaluated the effects of low-dose pirfenidone on IPF disease progression and patient survival in the real-world.

**Methods:**

This retrospective, observational study enrolled IPF patients seen at the time of diagnosis at a single center from 2008 to 2018. Longitudinal clinical and laboratory data were prospectively collected. We compared the clinical characteristics, survival, and pulmonary function decline between patients treated and untreated with various dose of pirfenidone.

**Results:**

Of 295 IPF patients, 100 (33.9%) received pirfenidone and 195 (66.1%) received no antifibrotic agent. Of the 100 patients who received pirfenidone, 24 (24%), 50 (50%), and 26 (26%), respectively, were given 600, 1200, and 1800 mg pirfenidone daily. The mean survival time was 57.03 ± 3.90 months in the no-antifibrotic drug group and 73.26 ± 7.87 months in the pirfenidone-treated group (p = 0.027). In the unadjusted analysis, the survival of the patients given pirfenidone was significantly better (hazard ratio [HR] = 0.69, 95% confidence interval [CI]: 0.48–0.99, p = 0.04). After adjusting for age, gender, body mass index, and the GAP score [based on gender (G), age (A), and two physiological lung parameters (P)], survival remained better in the patients given pirfenidone (HR = 0.56, 95% CI: 0.37–0.85, p = 0.006). In terms of pulmonary function, the decreases in forced vital capacity (%), forced expiratory volume in 1 s (%) and the diffusing capacity of lung for carbon monoxide (%) were significantly smaller (p = 0.000, p = 0.001, and p = 0.007, respectively) in patients given pirfenidone.

**Conclusions:**

Low-dose pirfenidone provided beneficial effects on survival and pulmonary function decline in the real-world practice.

## Introduction

Idiopathic pulmonary fibrosis (IPF) is a chronic, progressive fibrosing interstitial pneumonia of unknown etiology [1]. IPF is fatal, characterized by progressive dyspnea and irreversible loss of lung function. The disease course is variable and unpredictable, and the median survival time from diagnosis is 2–4 years [2]. The pathophysiology of IPF is characterized by recurrent epithelial cell injury, senescence of alveolar epithelial cells, the expression of profibrotic mediators that stimulate matrix deposition by myofibroblasts, microbiome changes, and abnormalities in host defense [3]. Two anti-fibrotic drugs, pirfenidone and nintedanib, are used to effectively and safely treat IPF [4]. Pirfenidone is an orally administered pyridine with anti-inflammatory, anti-oxidant, and anti-fibrotic actions. The drug inhibits collagen synthesis, downregulates of expression of tumor growth factor (TGF)-β and tumor necrosis factor (TNF)-α, and reduces fibroblast proliferation [5]. In the CAPACITY and ASCEND trials, pirfenidone at 2403 mg/day (801 mg three times daily) reduced disease progression reflected in lung function, exercise tolerance, and progression-free survival, in IPF patients compared to a placebo [6, 7]. Pirfenidone was approved in Europe in 2011, and in the United States in 2014. The most recent clinical practice guideline for IPF includes conditional recommendations for its use in most patients [4]. The most common adverse events (AEs) observed in clinical trials and the real-world experience are gastrointestinal (GI) and skin-related; they are generally mild-to-moderate and rarely lead to treatment discontinuation [6–10]. Although long-term pirfenidone is generally well-tolerated, dose modification can reduce both the incidence and severity of AEs, and promote patient compliance [11, 12].

As a complement to clinical trials, real-world studies have less strict inclusion criteria [13]. Several studies have evaluated the real-world efficacy and safety of pirfenidone, which was found to delay disease progression [8, 13, 14]. In a phase III clinical trial, Japanese patients given low-dose pirfenidone (1200 mg/day) showed a significantly slower decline in forced vital capacity (FVC) compared to the placebo group [9]. Although real-world data are gradually accumulating, more longitudinal data on IPF disease progression and survival in patients on low-dose pirfenidone (≤1200 mg/day) are required [8]. This study compared the clinical characteristics, overall survival (OS), and pulmonary function decline between patients treated and untreated with low-dose pirfenidone at a single institution.

## Methods

### Study design and population

This single-center, retrospective observational study reviewed the medical records and laboratory test data of patients diagnosed with IPF at Bucheon St. Mary’s hospital, South Korea. IPF patients consecutively enrolled at diagnosis from 2008 to 2018 were evaluated. Patients’ characteristics (age, gender, smoking status, BMI) and clinical characteristics (medical history, diagnosis, pulmonary function, radiologic patterns, biomarkers) were collected. Clinical and laboratory data, including pulmonary function test and image studies were collected regularly and in real-time at the time of workup and follow up by the pre-set protocols specified for ILD. All the data collected were again retrospectively reviewed. Data on medications were collected throughout the study, including immunosuppressive agents.

The radiological images and pathological findings were evaluated by pulmonologists, radiologists, and pathologists. All IPF diagnoses were reconfirmed according to the Official American Thoracic Society (ATS)/European Respiratory Society (ERS)/Japanese Respiratory Society (JRS)/Latin American Thoracic Association (ALAT) Clinical practice guideline, 2018 [1]. The GAP index and a staging system are used to predict the clinical prognosis of IPF patients. The GAP index evaluates gender (G), age (A), and two physiological lung parameters (P), i.e., the FVC and diffusing capacity of lung for carbon monoxide (DLCO). Patients were staged as I–III, for which the estimated 1-year mortality rates are 5.6%, 16.2% and 39.2%, respectively [15].

There is a compulsory and universal health insurance system in South Korea. Pirfenidone is an expensive drug and was approved by the health insurance system in October 2015. The reimbursement criteria for pirfenidone are strict and are limited to patients with a definite IPF based on high resolution CT and/or surgical lung biopsy with FVC ≤ 90% or DLCO ≤ 80%. Therefore, since then, pirfenidone has been established as the standard-of-care for patients who satisfy the above criteria, and is also used in our hospital. However, nintedanib is not approved to reimburse and is rarely prescribed due to its high cost in South Korea.

Pirfenidone was initially administered with food as three daily 200 mg doses, and then gradually increased to the full dose of 1800 mg/day over every 2–4 weeks in daily increments of 200–600 mg. The patient’s condition was carefully monitored during this process. Patients who could not tolerate the full recommended dose (1800 mg/day) due to AEs were assigned to the low-dose (600 or 1200 mg/day) pirfenidone group. Dose escalation, dose reduction, or discontinuation of pirfenidone was made at the physician’s discretion, considering the patients’ condition and not for research purposes.

### Statistical analysis

Data were expressed as the mean ± standard deviation (SD) or the mean ± standard error (SE), or as numbers with percentages, as appropriate. A student’s *t*-test was used to compare continuous variables between the groups, and the Pearson chi-squared test or ANOVA was used to compare of categorical variables. Survival probabilities were estimated using the Kaplan-Meier method. Adjusted hazard ratios (aHRs) and 95% confidence intervals (CIs) were calculated using a Cox proportional hazards model adjusted for age, gender, body mass index (BMI), and the GAP score. The statistical analyses were performed using SAS software (ver. 9.4; SAS Institute, Cary, NC, USA), except for the survival analysis, which was done using SPSS Statistics for Windows software (ver. 24.0; IBM Corp., Armonk, NY, USA). A two-sided p-value ≤ 0.05 was taken to indicate a significant difference.

### Ethics statement

The study was approved by the Institutional Review Board (IRB) and Ethics Committee of Bucheon St. Mary’s Hospital (IRB No.: 2021-3027-0001). The need for written informed consent was waived because of the retrospective design.

## Results

### Demographics

Baseline subject characteristics are presented in Table 1. Of the 295 patients diagnosed with IPF, 195 did not use any antifibrotic drug and 100 were taking pirfenidone. The mean age was 70.81 ± 10.68 years in the no-antifibrotic drug group and 68.87 ± 8.48 years in the pirfenidone-treated group (p = 0.099). The proportions of males (81.0% vs. 61.5%, p = 0.0007) and current or former smokers (75.0% vs. 57.4%, p = 0.003) were higher in the pirfenidone-treated group than in the no-antifibrotic drug group; there was no significant group difference in BMI (23.53 ± 3.35 vs. 23.71 ± 3.49 kg/m^2^, p = 0.675).

**Table 1:** Baseline epidemiological and clinical characteristics of the enrolled patients.

Characteristics	No-antifibrotic drug (n = 195)	Pirfenidone (n = 100)	p-value
Age, year	70.81 ± 10.68	68.87 ± 8.48	0.099
Male, n (%)	120 (61.5%)	81 (81.0%)	0.0007
BMI, kg/m^2^	23.71 ± 3.49	23.53 ± 3.35	0.675
Current or former smokers, n (%)	112 (57.4%)	75 (75.0%)	0.003
Pack years	21.44 ± 24.9	25.65 ± 21.01	0.151
Bronchoalveolar lavage (BAL), n (%)	94 (48.2%)	71 (71.0%)	0.001
Surgical lung biopsy, n (%)	44 (22.6%)	12 (12.0%)	0.029
6 minute walk test (6MWT), n (%)	368 ± 179.27	391.72 ± 157.47	0.411
SpO2 ≥90% after 6MWT, n (%)	153 (78.5%)	42 (42.0%)	0.0001
GAP score	2.91 ± 1.25	3.27 ± 1.35	0.0251
Stage, n (%)			0.242
I	144 (73.9%)	65 (65.0%)	
II	43 (22.0%)	31 (31.0%)	
III	8 (4.1%)	4 (4.0%)	
Chest CT pattern, n (%)			
UIP	140 (71.8%)	70 (70.0%)	
Probable UIP	31 (15.9%)	23 (23.0%)	
Indeterminate UIP	24 (12.3%)	7 (7.0%)	
Pulmonary function test (PFT)			
FVC (L)	2.54 ± 0.83	2.70 ± 0.76	0.122
FVC (% predicted)	81.73 ± 18.97	79.84 ± 18.99	0.435
FEV1 (L)	2.04 ± 0.63	2.21 ± 0.60	0.039
FEV1 (% predicted)	96.2 ± 24.74	95.35 ± 25.03	0.789
FEV1/FVC	81.49 ± 8.79	82.35 ± 7.02	0.377
DLCO (mL/mmHg/min)	11.13 ± 5.47	10.74 ± 4.46	0.536
DLCO (% predicted)	67.23 ± 24.95	64.01 ± 24.75	0.314

Values are expressed as mean ± standard deviation (SD).

Abbreviations: Standard deviation, SD; Body mass index, BMI; Bronchoalveolar lavage, BAL; 6-minute walk test (6MWT), Usual interstitial pneumonia, UIP; Pulmonary function test, PFT; Forced vital capacity, FVC; Forced expiratory volume in one second, FEV1; Diffusing capacity of lung for carbon monoxide, DLCO.

The forced expiratory volume in 1 second (FEV1) was significantly higher in the pirfenidone-treated group than the no-antifibrotic drug group (2.21 ± 0.60 vs. 2.04 ± 0.63 L, p = 0.039). Other pulmonary function parameters, including the FVC, FEV1/FVC ratio, and DLCO did not differ between the two groups.

The distance covered in the 6-minute walk test (6MWT) did not differ between the no-antifibrotic drug and pirfenidone-treated groups (368.0 ± 179.27 vs. 391.72 ± 157.47 m, p = 0.411), but the proportion of patients with a saturation of percutaneous oxygen (SpO2) percentage over 90% after the 6MWT was significantly higher in the former group (78.5% vs. 42.0%, p = 0.025).

The severity of IPF, as evaluated by the GAP index, was greater in the pirfenidone-treated than the no-antifibrotic drug group (3.27 ± 1.35 vs. 2.91 ± 1.25, p = 0.025). However, there was no significant difference between the two groups in the proportions of GAP stage I–III patients. The proportion of patients who underwent bronchoalveolar lavage (BAL) at the time of diagnosis was significantly higher in the pirfenidone-treated than no-antifibrotic drug group (71.0% vs. 48.2%, p = 0.001), while the proportion diagnosed with IPF via surgical lung biopsy was significantly higher in the latter group (12.0% vs. 22.6%, p = 0.029).

Of the 100 patients treated with pirfenidone, 24 received 600 mg/day (24.0%), 50 received 1200 mg/day (50.0%), and 26 received 1800 mg/day (26.0%) (Table 2). The mean age (70.12 ± 8.90, 70.52 ± 8.10 and 65.00 ± 7.91 years, respectively, p = 0.021) was significantly lower, and the BMI was significantly higher (22.04 ± 3.04, 23.49 ± 2.95 and 24.99 ± 3.81 kg/m^2^, respectively, p = 0.007), in the group receiving 1800 mg/day of pirfenidone.

**Table 2:** Baseline epidemiological and clinical characteristics according to dose in patients treated with pirfenidone (n = 100).

Characteristics	Pirfenidone 600 mg/day (n = 24)	Pirfenidone 1200 mg/day (n = 50)	Pirfenidone 1800 mg/day (n = 26)	p-value
Age, year	70.12 ± 8.90	70.52 ± 8.10	65.00 ± 7.91	0.021
Male, n (%)	18 (75.0%)	40 (80.0%)	23 (88.5%)	0.925
BMI, kg/m^2^	22.04 ± 3.04	23.49 ± 2.95	24.99 ± 3.81	0.007
BSA, m^2^	1.60 ± 0.21	1.67 ± 0.25	1.74 ± 0.18	0.037
Current or former smokers, n (%)	18 (75.0%)	35 (70.0%)	22 (84.6%)	0.377
Pack years	25.36 ± 20.85	24.88 ± 22.96	27.44 ± 17.59	0.884
GAP score	3.5 ± 1.47	3.36 ± 1.16	2.88 ± 1.56	0.223
Stage, n (%)				0.386
I	13 (54.2%)	33 (66.0%)	19 (73.1%)	
II	10 (41.7%)	16 (32.0%)	5 (19.2%)	
III	1 (4.1%)	1 (2.0%)	2 (7.7%)	
Pulmonary function test (PFT)				
FVC (L)	2.66 ± 0.97	2.65 ± 0.74	2.83 ± 0.60	0.619
FVC (% predicted)	81.23 ± 22.26	79.88 ± 20.00	78.62 ± 14.10	0.895
FEV1 (L)	2.14 ± 0.78	2.19 ± 0.58	2.31 ± 0.49	0.593
FEV1 (% predicted)	96.82 ± 30.11	96.88 ± 26.74	91.31 ± 15.88	0.632
FEV1/FVC	80.82 ± 6.83	83.21 ± 6.91	82.08 ± 7.42	0.410
DLCO (mL/mmHg/min)	9.95 ± 5.16	10.40 ± 4.06	12.04 ± 4.42	0.205
DLCO (% predicted)	60.09 ± 23.76	64.23 ± 25.28	66.92 ± 25.11	0.637

Values are expressed as the mean ± standard deviation (SD).

Abbreviations: Standard deviation, SD; Body mass index, BMI; Body surface area, BSA; Pulmonary function test, PFT; Forced vital capacity, FVC; Forced expiratory volume in one second, FEV1; Diffusing capacity of lung for carbon monoxide, DLCO.

### Survival analysis

**Fig 1:**
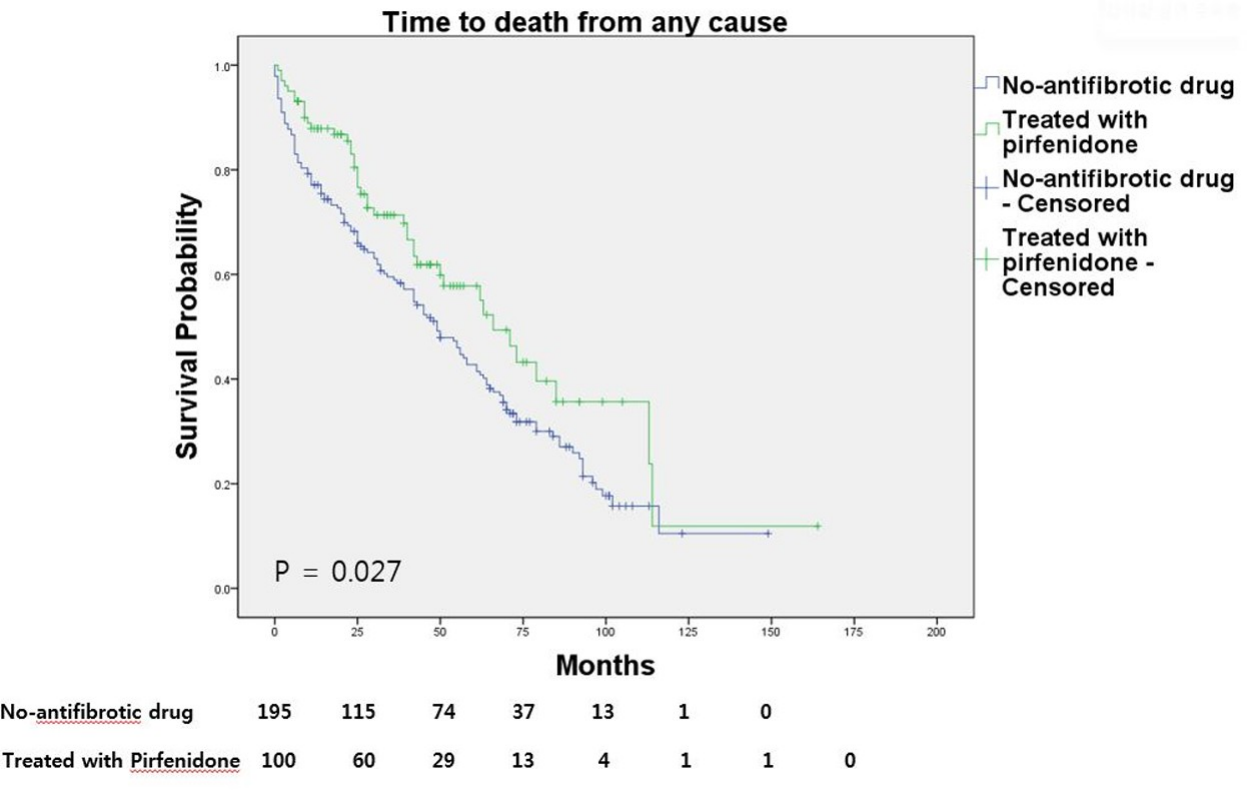
Overall survival of IPF patients on pirfenidone and no-antifibrotic treatment.

**Fig 2:**
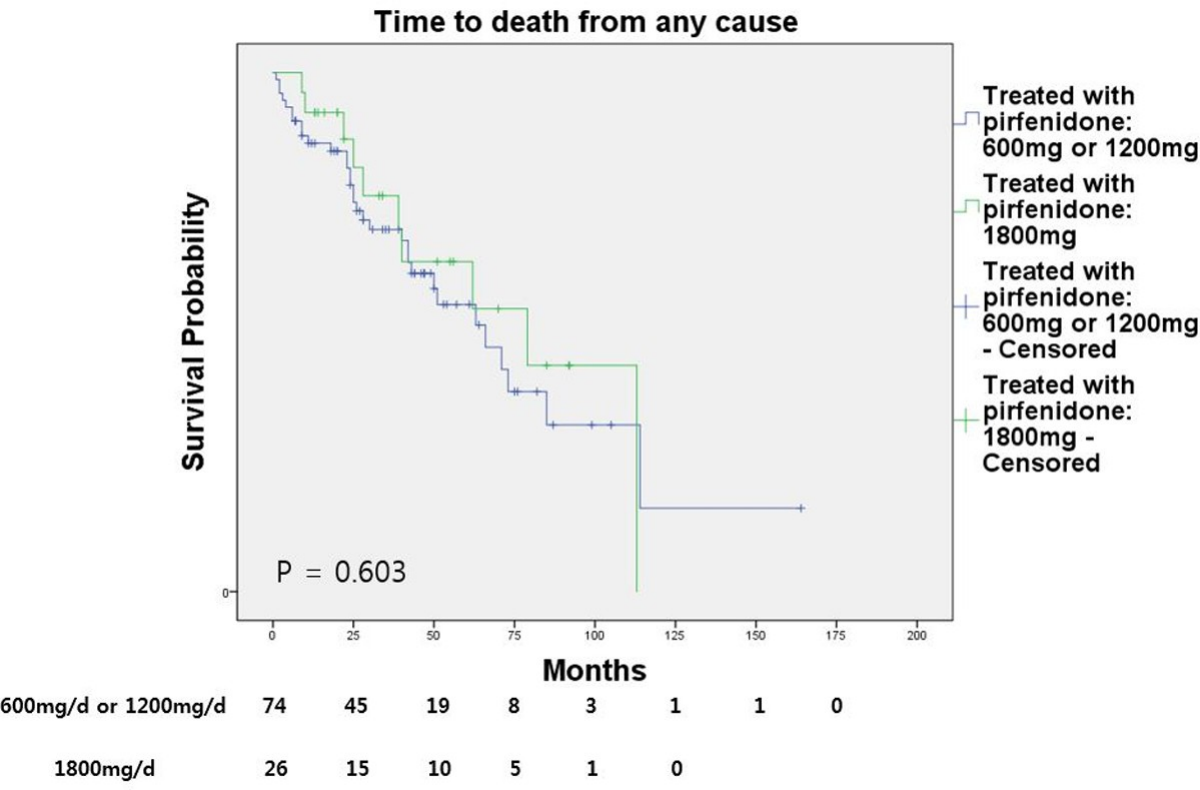
Overall survival of IPF patients according to the pirfenidone dose–full recommended dose of 1800 mg/day vs. relatively low-dose of 1200 mg/day or less.

The mean OS was 57.03 ± 3.90 months in the no-antifibrotic drug group and 73.26 ± 7.87 months in the pirfenidone-treated group (p = 0.027; Fig 1). There was no significant difference in OS between patients given the full dose of pirfenidone recommended in South Korea (1800 mg/day) and those treated with lower doses (600 or 1200 mg/day) (Fig 2). The mean survival time was 73.26 ± 10.12 and 72.96 ± 9.75 months in patients treated with the full and lower doses, respectively (p = 0.603).

The association between pirfenidone treatment and mortality was analyzed (Table 3). Regardless of dose, the mortality rate was significantly lower in patients on pirfenidone (HR = 0.691, 95% CI: 0.484–0.986, p = 0.042). The analysis was repeated after adjusting for age, gender, BMI, and the GAP score; the mortality rate of patients treated with pirfenidone remained significantly lower (HR = 0.563, 95% CI: 0.374–0.845, p = 0.006). There was no significant association between the dose of pirfenidone (600 or 1200 vs. 1800 mg/day) and mortality (HR = 0.865, 95% CI: 0.421–1.779, p = 0.694), including adjusting for age, gender, BMI, and the GAP score (HR = 1.050, 95% CI: 0.472–2.338, p = 0.905).

**Table 3:** Effects of pirfenidone treatment on mortality using Cox proportional hazard regression model.

	Unadjusted analysis			Adjusted analysis		
	HR	95% CI	P value	HR	95% CI	P value
No-antifibrotic drug	1			1		
Treated with pirfenidone	0.691	0.484–0.986	0.042	0.563	0.374–0.845	0.006
Relatively low dose pirfenidone (600mg/d and 1200mg/d)	1			1		
Full recommended dose pirfenidone (1800mg/d)	0.865	0.421–1.179	0.694	1.050	0.472–2.338	0.905

Abbreviations: Hazard ratio, HR; Confidence interval, CI.

### Pulmonary function

Pulmonary function was analyzed in IPF patients who had undergone at least two pulmonary function tests (87 patients who did not use antifibrotic drugs and 55 patients on pirfenidone). Significantly smaller decreases in all pulmonary function indices, except the FEV1/FVC, were seen in patients treated with any dose (600, 1200, or 1800 mg/day) of pirfenidone than those not with the drug (Tables 4–6). There was no significant difference in all pulmonary function indices in patients treated with the full dose (1800 mg/day) of pirfenidone and lower dose (600 or 1200 mg/day) (Table 7).

**Table 4:** Comparison of pirfenidone treatment and annual decline in pulmonary function.

	Annual decline		
	No-antifibrotic drug (n = 87)	Treated with pirfenidone (n = 55)	p-value
ΔFVC (L)	-0.328 ± 0.301	-0.130 ± 0.367	0.001
ΔFVC (% predicted)	-9.85 ± 11.432	-1.548 ± 9.809	0.000
ΔFEV1 (L)	-0.219 ± 0.244	-0.032 ± 0.312	0.000
ΔFEV1 (% predicted)	-9.786 ± 13.917	1.880 ± 25.670	0.001
ΔFEV1/FVC	1.343 ± 5.841	3.297 ± 16.983	0.326
ΔDLCO (mL/mmHg/min)	-2.177 ± 2.975	-0.572 ± 2.741	0.002
ΔDLCO (% predicted)	-11.695 ± 16.819	0.765 ± 37.177	0.007

Values are expressed as the mean ± standard deviation (SD).

Abbreviations: Standard deviation, SD; Forced vital capacity, FVC; Forced expiratory volume in one second, FEV1; Diffusing capacity of lung for carbon monoxide, DLCO.

**Table 5:** Comparison of annual decline in pulmonary function between patients who were not treated with antifibrotic drugs and patients who were treated with full recommended dose of pirfenidone (1800mg/d).

	Annual decline		
	No-antifibrotic drug (n = 87)	Treated with full recommended dose of pirfenidone (1800mg/d) (n = 19)	p-value
ΔFVC (L)	-0.328 ± 0.301	-0.081 ± 0.287	0.002
ΔFVC (% predicted)	-9.85 ± 11.432	-2.123 ± 7.683	0.001
ΔFEV1 (L)	-0.219 ± 0.244	-0.042 ± 0.268	0.014
ΔFEV1 (% predicted)	-9.786 ± 13.917	-0.963 ± 10.225	0.003
ΔFEV1/FVC	1.343 ± 5.841	1.066 ± 4.062	0.807
ΔDLCO (mL/mmHg/min)	-2.177 ± 2.975	-0.793 ± 2.533	0.045
ΔDLCO (% predicted)	-11.695 ± 16.819	-2.921 ± 13.123	0.018

Values are expressed as the mean ± standard deviation (SD).

Abbreviations: Standard deviation, SD; Forced vital capacity, FVC; Forced expiratory volume in one second, FEV1; Diffusing capacity of lung for carbon monoxide, DLCO.

**Table 6:** Comparison of annual decline in pulmonary function between patients who were not treated with antifibrotic drugs and patients who were treated with relatively low dose of pirfenidone (600mg/d or 1200mg/d).

	Annual decline		
	No-antifibrotic drug (n = 87)	Treated with relatively low dose of pirfenidone (600mg/d or 1200mg/d) (n = 36)	p-value
ΔFVC (L)	-0.328 ± 0.301	-0.155 ± 0.405	0.010
ΔFVC (% predicted)	-9.85 ± 11.432	-1.245 ± 10.854	0.000
ΔFEV1 (L)	-0.219 ± 0.244	-0.028 ± 0.338	0.001
ΔFEV1 (% predicted)	-9.786 ± 13.917	3.380 ± 30.921	0.001
ΔFEV1/FVC	1.343 ± 5.841	4.471 ± 20.794	0.198
ΔDLCO (mL/mmHg/min)	-2.177 ± 2.975	-0.455 ± 2.873	0.004
ΔDLCO (% predicted)	-11.695 ± 16.819	2.711 ± 45.085	0.011

Values are expressed as the mean ± standard deviation (SD).

Abbreviations: Standard deviation, SD; Forced vital capacity, FVC; Forced expiratory volume in one second, FEV1; Diffusing capacity of lung for carbon monoxide, DLCO.

**Table 7:** Comparison of annual decline in pulmonary function between patients who were treated with full recommended dose of pirfenidone (1800mg/d) and relatively low dose of pirfenidone (600mg/d or 1200mg/d).

	Annual decline		
	Treated with relatively low dose of pirfenidone (600mg/d or 1200mg/d) (n = 36)	Treated with full recommended dose of pirfenidone (1800mg/d) (n = 19)	p-value
ΔFVC (L)	-0.155 ± 0.405	-0.081 ± 0.287	0.434
ΔFVC (% predicted)	-1.245 ± 10.854	-2.123 ± 7.683	0.730
ΔFEV1 (L)	-0.028 ± 0.338	-0.042 ± 0.268	0.868
ΔFEV1 (% predicted)	3.380 ± 30.921	-0.963 ± 10.225	0.447
ΔFEV1/FVC	4.471 ± 20.794	1.066 ± 4.062	0.348
ΔDLCO (mL/mmHg/min)	-0.455 ± 2.873	-0.793 ± 2.533	0.656
ΔDLCO (% predicted)	2.711 ± 45.085	-2.921 ± 13.123	0.490

Values are expressed as the mean ± standard deviation (SD).

Abbreviations: Standard deviation, SD; Forced vital capacity, FVC; Forced expiratory volume in one second, FEV1; Diffusing capacity of lung for carbon monoxide, DLCO.

### Adverse events

The AEs of patients on pirfenidone are summarized in Table 8. Most of the AEs affected the GI tract and skin. The proportion of patients who experienced at least one AE was significantly higher in the patient group treated with the full pirfenidone dose than the groups given the lower doses (44.6% vs. 92.3%, p = 0.000). However, the AE incidence, and the frequency and causes of pirfenidone discontinuation, did not differ between the two groups (Table 9). The proportion of patients with follow-up loss showed a tendency to be higher in the group of patients who were prescribed relatively low-dose pirfenidone. Still, the detailed reason could not be identified.

**Table 8:** Comparison of incidence of adverse events between patients who were treated with full recommended dose of pirfenidone (1800mg/d) and relatively low dose of pirfenidone (600mg/d or 1200mg/d).

	Treated with relatively low dose of pirfenidone (600mg/d or 1200mg/d) (n = 74)	Treated with full recommended dose of pirfenidone (1800mg/d) (n = 26)	p-value
≥1 AE of any type	33 (44.6%)	24 (92.3%)	0.000
Poor oral intake	18 (24.3%)	7 (26.9%)	0.792
Nausea, vomiting	9 (12.2%)	4 (15.4%)	0.674
Diarrhea	6 (8.1%)	0 (0.0%)	0.134
Dyspepsia	5 (6.8%)	4 (15.4%)	0.186
Skin rash, itching	7 (9.5%)	2 (7.7%)	0.787
Neurological disorder	3 (4.1%)	0 (0.0%)	0.297
General weakness	4 (5.4%)	0 (0.0%)	0.226

Abbreviations: Adverse events, AE.

**Table 9:** Comparison of cause of pirfenidone discontinuation between patients who were treated with full recommended dose of pirfenidone (1800mg/d) and relatively low dose of pirfenidone (600mg/d or 1200mg/d).

	Treated with relatively low dose of pirfenidone (600mg/d or 1200mg/d) (n = 74)	Treated with full recommended dose of pirfenidone (1800mg/d) (n = 26)	p-value
Discontinuation of any cause	47 (63.5%)	11 (42.3%)	0.059
Death	8 (10.8%)	5 (19.2%)	0.272
Follow up loss	21 (28.4%)	3 (11.5%)	0.084
Adverse events	11 (14.9%)	2 (7.7%)	0.350
IPF progression	12 (16.2%)	1 (3.8%)	0.107
Malignancy	2 (2.7%)	1 (3.8%)	0.769

## Discussion

In South Korea, pirfenidone was approved by the Korean food and Drug Administration in 2012. However, due to the high price and the lack of clinical practice of pirfenidone, it was not included in the health insurance system until October 2015. Among enrolled patients, the majority of patients not treated with pirfenidone were diagnosed in the pre-antifibrotic era.

In addition, the reimbursement criteria for pirfenidone are strict and are limited to patients with a definite usual interstitial pneumonia pattern on high resolution CT or IPF diagnosed by surgical lung biopsy and with FVC ≤ 90% or DLCO ≤ 80%. Among patients diagnosed with IPF, patients diagnosed earlier than 2015 and did not meet the above pulmonary function criteria because their pulmonary function was preserved cannot be treated with pirfenidone.

The Official ATS/ERS/JRS/ALAT Clinical practice guideline in 2015 recommended not use combination therapy of N-acetylcysteine, azathioprine, and prednisone in patients with IPF. Previously, immune suppression was considered important in the treatment of IPF [4]. In this study, there was no significant difference in the proportion of prednisone prescribed in 34.8% in the non-antifibrotic drug group and 36.0% in the pirfenidone-treated group (p = 0.858). Azathioprine was prescribed for only three patients in the non-antifibrotic drug group, not because IPF, but because of the treatment of inflammatory myopathy developed later. But these cases could not be clearly classified as CTD-ILD or IPF with combined CTD even after case-review. Only N-acetylcysteine had a significantly higher prescription rate in the no-antifibrotic drug group (50.9% vs. 29.0%, p = 0.001).

Although several randomized clinical trials and real-world studies have shown that pirfenidone is efficacious, the doses used were much higher than those employed in South Korea. Pirfenidone doses are often reduced in the real-world due to AEs. As it is unclear whether a lower dose is less effective than a higher one, many clinicians hesitate to prescribe lower pirfenidone doses.

In a Japanese phase III clinical trial, both the high-dose (1800 mg/day) and low-dose (1200 mg/day) pirfenidone groups exhibited improved FVC compared to a placebo group [9]. In the CAPACITY trial (Study 004), patients were assigned to a pirfenidone 2403 mg/day, pirfenidone 1197 mg/day, or placebo group in a 2:1:2 ratio. The 2403 mg/day dose was derived by normalizing of the 1800 mg/day dose used in Japanese studies accruing to the predicted body weights of a predominantly US-based population [7]. Pirfenidone at 2403 mg/day significantly reduced the mean decrease in the predicted FVC compared to placebo. The outcomes of the pirfenidone 1197 mg/day group were intermediate between those of the pirfenidone 2403 mg/day and placebo groups [7].

Pirfenidone and another antifibrotic, nintedanib, have become the gold standard for IPF treatment [4]. Pirfenidone is safe and tolerable in the long term. Nevertheless, pirfenidone-related AEs often lead to dose reduction and treatment interruption strategy, and a significant proportion of patients discontinue treatment [11]. In the CAPACITY (Study 004 and Study 006) and ASCEND trials, treatment was discontinued because of AEs in 15% and 14.4% of patients in the pooled pirfenidone groups, respectively [6, 7].

The above-mentioned phase III clinical trials served as the basis for expecting efficacy in relatively low-dose pirfenidone (1200 mg/day). However, the efficacy and safety of lower doses (≤ 1200 mg/day) have not been studied. Also, patients with advanced disease (FVC < 50% or DLCO < 30%) are excluded from clinical trials; real-world studies thus provide more informative data on the efficacy and safety of antifibrotic compounds. In fact, for five (5%) of our patients treated with pirfenidone, the FVC and DLCO were predicted to be below 50% and 30%, respectively. We studied the efficacy of relatively low-dose pirfenidone (≤ 1200 mg/day) in terms of OS and pulmonary function; the low dose was not inferior to the high dose.

Several studies have analyzed the effects of pirfenidone on the survival and all-cause mortality of IPF patients. When the ASCEND and CAPACITY populations were pooled, overall all-cause mortality was shown to be reduced by pirfenidone. At week 52, the all-cause mortality of the pirfenidone 2403 mg/day and placebo groups were 3.5% and 6.7%, respectively (p = 0.01) [6]. During post-hoc analysis of the trial data, patients with advanced IPF (FVC < 50% and/or DLCO < 35%) were analyzed; the all-cause mortality rates in the pirfenidone and placebo groups at week 52 were 4.4% and 15.0%, respectively (HR = 0.28, 95% CI: 0.09–0.86, p = 0.018) [16]. In the RECAP trial (based on the ASCEND and CAPACITY trials), the median survival time from the first dose of pirfenidone (2403 mg/day) was 77.2 months [10]. In a real-world Italian study, the 3-year survival of IPF patients taking pirfenidone was 73% [17]. In the Czech EMPIRE registry, the 60-month OS rates of pirfenidone and no-antifibrotic treatment groups were 0.559 (95% CI: 0.474–0.644) and 0.315 (95% CI: 0.234–0.396), respectively (p = 0.002) [18].

Although there was a difference in that both antifibrotic drugs were included, Cameli et al. including 139 patients treated with pirfenidone and 124 patients treated with nintedanib, according to the study, the median survival was 1224 days during an observational period of 885.3 ± 559.5 days, and there was no significant difference between the two drug groups [19].

In our study, the 1-year all-cause mortality rate was 12.1% in the pirfenidone group (any dose) and 22.9% in the no-antifibrotic drug group; these rates are higher than those in clinical trials. Similarly, the 3-year OS rates were 71.3% and 58.9% in the pirfenidone and no-antifibrotic drug groups, respectively; the respective 5-year OS rates were 57.8% and 42.8%. The mean survival of our pirfenidone-treated group was evaluated over a long period (73.26 ± 7.87 months). Also, most of the pirfenidone-treated patients received only 1200 mg/day of the drug, or less, in contrast to previous real-world studies; the low doses enhanced survival. To the best of our knowledge, no study has compared survival between patients on high- and low-dose pirfenidone.

In the non-antifibrotic drug group, the mean OS was 57.03 ± 3.90 months, which is high compared to other real-world studies. In South Korea, medical costs are relatively low due to the national health insurance. Even if there are no symptoms, low-dose chest CT is performed every year for lung cancer screening for ever smokers over the age of 55 and 30 pack years. In addition, access to CT is high, including cases where chest CT is performed due to other diseases such as pneumonia, tuberculosis, and COPD. Therefore, patients with IPF are often diagnosed early with no symptoms and preserved lung function. On the other side, the reimbursement criteria for pirfenidone are relatively strict, and eventually many patients with early IPF were included in the group who did not treat with pirfenidone.

In a Japanese clinical trial, the mean decrease in FVC over 52 weeks was 97, 15, and 169 mL in high-dose pirfenidone, low-dose pirfenidone, and placebo groups, respectively [20]. In the ASCEND trial, the FVC had decreased by 164 mL and 280 mL in pirfenidone and placebo groups, respectively, after 52 weeks (p < 0.001) [6]. Post-hoc analysis of patients with advanced IPF (FVC < 50% and/or DLCO < 35%) in the CAPACITY and ASCEND trials revealed that the annual decline in FVC was significantly smaller in the pirfenidone than placebo group (150 vs. 278 mL, p = 0.003) [16].

In a real-world study, Chaudhuri et al. measured the changes in FVC and DLCO each 6 months before and after pirfenidone commencement. The FVC decline changed from -1.043 ± 1.605 to -0.197 ± 0.231, and DLCO decline changed from -1.427 ± 1.568 to 0.1 ± 0.367 [21].

Song et al. showed that low-dose pirfenidone was effective in the real-world; the adjusted mean FVC decreased by 200.7, 88.4, and 94.7 mL in control, low-dose (< 1200 mg/day), and high-dose groups, respectively, in 1 year (p = 0.021) [22]. As in our study, there was no significant difference in the extent of the decrease in FVC between the low- and high-dose groups. However, unlike our study, survival was not analyzed by pirfenidone dose in the previous study.

We found that the annual decreases in FVC and DLCO were significantly smaller in the real-world when the recommended dose of pirfenidone was prescribed, and when the dose was 1200 mg/day or less. The annual decline of FEV1 was also significantly smaller, suggesting that FEV1 could serve as an indicator of pirfenidone efficacy.

In the FIBRONET study, the shorter the difference between the time of IPF diagnosis and the start of antifibrotic treatment, the higher the likelihood that the baseline lung function was preserved and the higher the possibility of relatively stable lung function after 12 months of observation [23].

In this study, most of the AEs involved the GI tract and skin, as in other real-world studies [14, 17, 22, 24]. Song et al. reported that GI AEs, such as dyspepsia, anorexia, and nausea, were significantly more common in low- than high-dose groups [22]. We found that the AE incidence did not vary by dose, but the proportion of patients who experienced at least one AE, of any type, was significantly higher in the group treated with the full recommended dose of pirfenidone (1800 mg/day). That group exhibited a significantly higher BMI compared to the low-dose groups (22.04 ± 3.04, 23.49 ± 2.95 and 24.99 ± 3.81 kg/m^2^ in the 600, 1200 and 1800 mg/day groups, respectively, p = 0.007); similar trends were reported by other studies [7, 22]. Fang et al. showed that patients with a BMI < 25 kg/m^2^ were at higher risk of disease progression, acute exacerbation, and death than overweight patients (BMI ≥ 25 kg/m^2^) [25]. A high BMI was associated with better nutritional status, which enhances the response to pharmacologic treatment and slows the disease course. In our study, although patients given low-dose pirfenidone had a low BMI, neither the OS nor pulmonary function decline were poorer. However, the fact that patients with higher BMI values were more tolerant of high-dose pirfenidone is in line with Fang et al. Thus, in some patients, depending on the BMI, dose reduction may be possible without any reduction in efficacy.

Uehara et al. classified the patients into two groups based on the median value of body surface area (BSA) adjusted dose of pirfenidone (876 mg/m^2^) [26]. The patient group taking the higher dose of pirfenidone (≥876 mg/m^2^) showed a lower decline in lung function (Δ%FVC) compared to the patient group taking the lower adjusted dose (<876 mg/m^2^). However, a significantly higher BSA-adjusted dose was found in patients with AE, and most patients who discontinued pirfenidone had received a higher dose of pirfenidone. In particular, pirfenidone at medium doses (876–1085 mg/m^2^) showed a significantly lower annual decline in %FVC than patients taking lower doses, as well as significantly reduced AE, resulting in long term effective treatment. In the present study, there was a significant difference in BSA by pirfenidone dose in patients treated with pirfenidone (1.60 ± 0.21, 1.67 ± 0.25, and 1.74 ± 0.18 m^2^ in the 600, 1200, and 1800 mg/day groups, respectively, p = 0.037, Table 2). When applying the above criteria to our patients, all patients with a higher BSA-adjusted dose of pirfenidone (≥876 mg/m^2^) belong to the group receiving 1800 mg/day. But our study is not designed for the issue of BSA and dose and we cannot sure the relationship between BSA-adjusted dose and effectiveness. In South Korea, the dose of pirfenidone per pill is 200 mg, and in actual clinical practice, it isn’t easy to adjust the dose between 1200 mg/day and 1800 mg/day. Therefore, we tried to show that pirfenidone can also be used efficaciously and safely at a relatively lower dose of 1200 mg/day or less.

Our study had several limitations. First, it used a retrospective design and was conducted at a single institution. Therefore, selection bias and an influence of unknown confounding factors cannot be ruled out. Second, as this was an observational study, it was difficult to obtain progression-free survival data because only a few pulmonary function tests were repeated, at irregular intervals. Finally, we did not explore acute exacerbations, which could contribute to mortality and may be important during the clinical course of IPF patients.

## Conclusions

Low-dose pirfenidone provided beneficial effects on survival and pulmonary function decline in real-world practice. We suggests that continuation of the medication, even at low doses, can be beneficial for IPF patients. Physicians should consider dose reduction rather than discontinuation if the patient’s condition permits this.

## Supporting information

S1 Data (XLSX)

## Abbreviations

IPF: idiopathic pulmonary fibrosis
HR: hazard ratio
CI: confidence interval
BMI: body mass index
FVC: forced vital capacity
FEV1: forced expiratory volume in 1 second
DLCO: diffusing capacity of lung for carbon monoxide
TGF-β: tumor growth factor-β
TNF-α: tumor necrosis factor-α
AEs: adverse events
GI: gastrointestinal
OS: overall survival
ATS: American Thoracic Society
ERS: European Respiratory Society
JRS: Japanese Respiratory Society
ALAT: Latin American Thoracic Association
GAP: gender (G), age (A), and two physiological lung parameters (P), i.e., the FVC and DLCO
SD: standard deviation
SE: standard error
6MWT: 6-minute walk test
SpO2: saturation of percutaneous oxygen
BAL: bronchoalveolar lavage
BSA: Body surface area

## References

[1] Raghu G, Remy-Jardin M, Myers JL, Richeldi L, Ryerson CJ, Lederer DJ, et al. Diagnosis of Idiopathic Pulmonary Fibrosis. An Official ATS/ERS/JRS/ALAT Clinical Practice Guideline. Am J Respir Crit Care Med. 2018;198(5):e44–e68. Epub 2018/09/01. doi: 10.1164/rccm.201807-1255ST . 30168753

[2] Richeldi L, Collard HR, Jones MG. Idiopathic pulmonary fibrosis. The Lancet. 2017;389(10082):1941–52. doi: 10.1016/s0140-6736(17)30866-8 28365056

[3] Lederer DJ, Martinez FJ. Idiopathic Pulmonary Fibrosis. N Engl J Med. 2018;378(19):1811–23. Epub 2018/05/10. doi: 10.1056/NEJMra1705751 . 29742380

[4] Raghu G, Rochwerg B, Zhang Y, Garcia CA, Azuma A, Behr J, et al. An Official ATS/ERS/JRS/ALAT Clinical Practice Guideline: Treatment of Idiopathic Pulmonary Fibrosis. An Update of the 2011 Clinical Practice Guideline. Am J Respir Crit Care Med. 2015;192(2):e3–19. Epub 2015/07/16. doi: 10.1164/rccm.201506-1063ST . 26177183

[5] Kolb M, Bonella F, Wollin L. Therapeutic targets in idiopathic pulmonary fibrosis. Respir Med. 2017;131:49–57. Epub 2017/09/28. doi: 10.1016/j.rmed.2017.07.062 . 28947042

[6] King TE Jr., Bradford WZ, Castro-Bernardini S, Fagan EA, Glaspole I, Glassberg MK, et al. A phase 3 trial of pirfenidone in patients with idiopathic pulmonary fibrosis. N Engl J Med. 2014;370(22):2083–92. Epub 2014/05/20. doi: 10.1056/NEJMoa1402582 . 24836312

[7] Noble PW, Albera C, Bradford WZ, Costabel U, Glassberg MK, Kardatzke D, et al. Pirfenidone in patients with idiopathic pulmonary fibrosis (CAPACITY): two randomised trials. The Lancet. 2011;377(9779):1760–9. doi: 10.1016/S0140-6736(11)60405-4 21571362

[8] Cottin V, Maher T. Long-term clinical and real-world experience with pirfenidone in the treatment of idiopathic pulmonary fibrosis. Eur Respir Rev. 2015;24(137):545. Epub 2015/09/02. doi: 10.1183/09059180.50011514 . 25726556 PMC9487763

[9] Taniguchi H, Ebina M, Kondoh Y, Ogura T, Azuma A, Suga M, et al. Pirfenidone in idiopathic pulmonary fibrosis. Eur Respir J. 2010;35(4):821–9. Epub 2009/12/10. doi: 10.1183/09031936.00005209 . 19996196

[10] Costabel U, Albera C, Lancaster LH, Lin CY, Hormel P, Hulter HN, et al. An Open-Label Study of the Long-Term Safety of Pirfenidone in Patients with Idiopathic Pulmonary Fibrosis (RECAP). Respiration. 2017;94(5):408–15. Epub 2017/09/13. doi: 10.1159/000479976 . 28898890

[11] Lancaster LH, de Andrade JA, Zibrak JD, Padilla ML, Albera C, Nathan SD, et al. Pirfenidone safety and adverse event management in idiopathic pulmonary fibrosis. Eur Respir Rev. 2017;26(146). Epub 2017/12/08. doi: 10.1183/16000617.0057-2017 . 29212837 PMC9488585

[12] Nathan SD, Lancaster LH, Albera C, Glassberg MK, Swigris JJ, Gilberg F, et al. Dose modification and dose intensity during treatment with pirfenidone: analysis of pooled data from three multinational phase III trials. BMJ Open Respir Res. 2018;5(1):e000323. Epub 2018/08/18. doi: 10.1136/bmjresp-2018-000323 ; PubMed Central PMCID: PMC6089326. 30116539 PMC6089326

[13] Harari S, Caminati A. Idiopathic pulmonary fibrosis: from clinical trials to real-life experiences. Eur Respir Rev. 2015;24(137):420–7. Epub 2015/09/02. doi: 10.1183/16000617.0042-2015 . 26324803 PMC9487689

[14] Vancheri C, Sebastiani A, Tomassetti S, Pesci A, Rogliani P, Tavanti L, et al. Pirfenidone in real life: A retrospective observational multicentre study in Italian patients with idiopathic pulmonary fibrosis. Respir Med. 2019;156:78–84. Epub 2019/08/25. doi: 10.1016/j.rmed.2019.08.006 . 31445389

[15] Ley B, Ryerson CJ, Vittinghoff E, Ryu JH, Tomassetti S, Lee JS, et al. A multidimensional index and staging system for idiopathic pulmonary fibrosis. Ann Intern Med. 2012;156:684–91. doi: 10.7326/0003-4819-156-10-201205150-00004 22586007

[16] Nathan SD, Costabel U, Albera C, Behr J, Wuyts WA, Kirchgaessler KU, et al. Pirfenidone in patients with idiopathic pulmonary fibrosis and more advanced lung function impairment. Respir Med. 2019;153:44–51. Epub 2019/06/04. doi: 10.1016/j.rmed.2019.04.016 . 31153107

[17] Margaritopoulos GA, Trachalaki A, Wells AU, Vasarmidi E, Bibaki E, Papastratigakis G, et al. Pirfenidone improves survival in IPF: results from a real-life study. BMC Pulm Med. 2018;18(1):177. Epub 2018/11/25. doi: 10.1186/s12890-018-0736-z ; PubMed Central PMCID: PMC6251092. 30470213 PMC6251092

[18] Zurkova M, Kriegova E, Kolek V, Lostakova V, Sterclova M, Bartos V, et al. Effect of pirfenidone on lung function decline and survival: 5-yr experience from a real-life IPF cohort from the Czech EMPIRE registry. Respir Res. 2019;20(1):16. Epub 2019/01/23. doi: 10.1186/s12931-019-0977-2 ; PubMed Central PMCID: PMC6341650. 30665416 PMC6341650

[19] Cameli P, Refini RM, Bergantini L, d’Alessandro M, Alonzi V, Magnoni C, et al. Long-Term Follow-Up of Patients With Idiopathic Pulmonary Fibrosis Treated With Pirfenidone or Nintedanib: A Real-Life Comparison Study. Front Mol Biosci. 2020;7:581828. Epub 2020/10/27. doi: 10.3389/fmolb.2020.581828 ; PubMed Central PMCID: PMC7498677. 33102528 PMC7498677

[20] Taguchi Y, Ebina M, Hashimoto S, Ogura T, Azuma A, Taniguchi H, et al. Efficacy of pirfenidone and disease severity of idiopathic pulmonary fibrosis: Extended analysis of phase III trial in Japan. Respir Investig. 2015;53(6):279–87. Epub 2015/11/02. doi: 10.1016/j.resinv.2015.06.002 . 26521105

[21] Chaudhuri N, Duck A, Frank R, Holme J, Leonard C. Real world experiences: pirfenidone is well tolerated in patients with idiopathic pulmonary fibrosis. Respir Med. 2014;108(1):224–6. Epub 2013/11/26. doi: 10.1016/j.rmed.2013.11.005 . 24269005

[22] Song MJ, Moon SW, Choi JS, Lee SH, Lee SH, Chung KS, et al. Efficacy of low dose pirfenidone in idiopathic pulmonary fibrosis: real world experience from a tertiary university hospital. Sci Rep. 2020;10(1):21218. Epub 2020/12/06. doi: 10.1038/s41598-020-77837-x ; PubMed Central PMCID: PMC7719184. 33277557 PMC7719184

[23] Poletti V, Vancheri C, Albera C, Harari S, Pesci A, Metella RR, et al. Clinical course of IPF in Italian patients during 12 months of observation: results from the FIBRONET observational study. Respir Res. 2021;22(1):66. Epub 2021/02/26. doi: 10.1186/s12931-021-01643-w ; PubMed Central PMCID: PMC7903602. 33627105 PMC7903602

[24] Chung MP, Park MS, Oh IJ, Lee HB, Kim YW, Park JS, et al. Safety and Efficacy of Pirfenidone in Advanced Idiopathic Pulmonary Fibrosis: A Nationwide Post-Marketing Surveillance Study in Korean Patients. Adv Ther. 2020;37(5):2303–16. Epub 2020/04/17. doi: 10.1007/s12325-020-01328-8 ; PubMed Central PMCID: PMC7467484. 32297284 PMC7467484

[25] Fang C, Huang H, Guo J, Ferianc M, Xu Z. Real-world experiences: Efficacy and tolerability of pirfenidone in clinical practice. PLoS One. 2020;15(1):e0228390. Epub 2020/01/31. doi: 10.1371/journal.pone.0228390 ; PubMed Central PMCID: PMC6992179. 31999801 PMC6992179

[26] Uehara M, Enomoto N, Oyama Y, Suzuki Y, Kono M, Furuhashi K, et al. Body size-adjusted dose analysis of pirfenidone in patients with interstitial pneumonia. Respirology. 2018;23(3):318–24. Epub 2017/08/30. doi: 10.1111/resp.13145 . 28851013

